# A survey on the use of association rules mining techniques in textual social media

**DOI:** 10.1007/s10462-022-10196-3

**Published:** 2022-05-12

**Authors:** Jose A. Diaz-Garcia, M. Dolores Ruiz, Maria J. Martin-Bautista

**Affiliations:** Department of Computer Science and Artificial Intelligence, University of Granada, Granada, Spain

**Keywords:** Social media mining, Association rules, Text mining, Social networks

## Abstract

The incursion of social media in our lives has been much accentuated in the last decade. This has led to a multiplication of data mining tools aimed at obtaining knowledge from these data sources. One of the greatest challenges in this area is to be able to obtain this knowledge without the need for training processes, which requires structured information and pre-labelled datasets. This is where unsupervised data mining techniques come in. These techniques can obtain value from these unstructured and unlabelled data, providing very interesting solutions to enhance the decision-making process. In this paper, we first address the problem of social media mining, as well as the need for unsupervised techniques, in particular association rules, for its treatment. We follow with a broad overview of the applications of association rules in the domain of social media mining, specifically, their application to the problems of mining textual entities, such as tweets. We also focus on the strengths and weaknesses of using association rules for solving different tasks in textual social media. Finally, the paper provides a perspective overview of the challenges that association rules must face in the next decade within the field of social media mining.

## Introduction

The recent incursion of online social networks in our world has changed the economic and social paradigm of society. Thanks to social networks and social media, we can communicate with our relatives thousands of miles away, buy products, give our opinion on whether the product is good or not, be updated in real time or simply be entertained. 

The volume of information circulating on social networks daily has increased considerably and will continue to do so in a massive way. This has awakened the interest of countless small and large companies, research institutes and governments. These institutions have seen, in the massive processing of social media data, the opportunity to obtain competitive advantages or simply to improve the lives of citizens. Given its importance, many works in the field of Data Mining and Artificial Intelligence have emerged that are focused on analysing these sources of social network data. This has led to the emergence of a new area, known as social media mining.

Social media mining, which will be seen in detail in the next section, seeks to extract value from data from online social networks and social media. This is done by means of text mining techniques, machine learning, natural language processing, clustering, pattern mining and deep learning. In this paper, we focus on the application of association rules to the problem of social media mining. Association rules mining is a non-supervised data mining technique that has a growing protagonism nowadays. In the literature we find many surveys ( Adedoyin-Olowe et al. 2014), Nenkova and McKeown (2012) that try to address the problem with supervised approaches, usually classification (Liu and Zhang 2012). It is evident from the nature of the social media mining, that unsupervised techniques, such as association rules, have to be taken into account. This is because they provide a very interpretable solution, are able to deal with large amounts of unstructured data and do not need labelled datasets. This latter feature is very useful in online social networks where the volatility of the topics makes it difficult to have labelled data. 

Despite this, even recent surveys (Kumar and Jaiswal 2020) that address the use of data mining techniques in the field of user-generated content and sentiment analysis, neglect unsupervised techniques based on association rules. Therefore, we find it necessary to elaborate a review that addresses these techniques, which are so relevant nowadays due to their easily interpretable results and robustness in the absence of labelled data. Both problems are very relevant in the field of Big Data in social networks. As far as we know, this is the first survey that addresses social media mining with association rules. The paper also focuses on current challenges that are being addressed by the association rules and that open up promising avenues for future research. Thus, the major contributions of the survey to the state-of-the-art are:

- The review of current applications of association rules to the field of textual content in social networks that can set the groundwork for future work and applications.
- The identification of current challenges and future problems that need to be addressed in the coming years with association rules in user-generated content. These current challenges are a starting point for future studies.

The paper is organized as follows. The Sect. 2 explains the methodology followed by the literature review. In Sect. 3, social media mining problem is discussed, as well as the need for unsupervised techniques, such as association rules. In Sect. 4, we describe in detail different tasks of association rules in social media analysis. In Sect. 5, we present the field of application of the studied tasks. In Sect. 6, we look at the future challenges to be addressed by association rules, aiming to expose the main lines for future research. Section 7 offers a retrospective analysis of the survey and shows some statistics about the papers retrieved. Finally, in Sect. 8 we present the concluding remarks of our work.

## Methodology

The survey is based on journals and conferences in various spheres, providing a globalised vision of what the academic, business and social world is doing with the help of association rules. The methodology followed for the creation of the survey is very similar to the one proposed in the paper (Injadat et al. 2016), which is based on the methodology Systematic Literature Review (Budgen and Brereton 2006).

### Research questions

As far as we know, association rules are one of the main techniques used in Data Mining and are very popular in several sectors (Solanki and Patel 2015). In this review, our aim is to determine whether the field of social networks and textual content (microblogs, posts, tags...) is one of these sectors. Therefore, our research questions will be aimed at determining in which social network tasks and application domains we find solutions based on association rules. So, the research questions (RQs) that we aim to cover with the survey are:

- RQ1: What tasks are currently being solved with association rules in user-generated text?
- RQ2: What areas or fields of application have been addressed with association rules in user-generated text?
- RQ3: What are the current trends and future problems to be faced by association rules in social media mining?

### Search strategy

The search criteria employed has been based on the research questions and the main association rule mining algorithms. Concretely, using combinations of OR logical operators, we searched for articles that included the following terms in the abstract or the title of the paper: *association rules, pattern mining, Apriori, Eclat, FP growth* and *association rule mining.* In order to refine the search to the review domain, the OR combinations of the above words have been joined by an AND operator with the following terms: *Text mining, social media mining, social media, user-generated text* and *social networks*. The databases and search engines queried are those that encompass most JCR^1^ ranked journals and publications, which means that the value of the articles is proven with a rigorous peer review. We used the following databases:

- IEEE Xplore
- Google Scholar
- ScienceDirect
- Web of Science

### Study selection

The papers were selected based on the following inclusion criteria:

- Use association rules in social media.
- Scope of application is user-generated text on social networks.
- Studies indexed in at least one of the above databases.
- If the paper has different versions, we consider the most recent one.
- Papers published between the year 2000 and 2020. They correspond to the year in which social networks and blogs started to gain popularity (2000) and the last expired year (2020).

On the other hand, some articles were left out of the review based on the following exclusion criteria:

- Objectives of the paper were not well defined or the application filed was not clearly related to the user-generated text.
- Papers that apply association rules on non-user-generated textual content, e.g. on graphs.
- Theoretical studies on new association rule techniques that only name possible applications.

The papers were analysed manually, initially reading the abstract and checking whether they passed the inclusion criteria. In total, 43 papers were considered to pass the filter. Once they passed this cut-off, an exhaustive reading was carried out focusing on the association rules technique used, the task in which it is applied and the scope of application.

## The social media mining problem

Social media mining, according to Gundecha and Liu (2012), involves the process of representing, analysing and extracting meaningful and valuable social media patterns from data. In a latter stage, these patterns can be used in the decision-making processes of small or large companies. Social media mining is therefore a multidisciplinary field and its scope can be divided, according to Gundecha and Liu (2012), into the following areas of application:

- Community analysis: By means of graph theory (Yang et al. 2011) and clustering (Alanezi et al. 2021), communities within a target population are obtained. These can be users with similar interests, likes or preferences.
- Collaborative recommendation systems: The recommendation system is based on the assumption that similar users will have similar likes, so that recommendation systems can be refined to take these factors into account. In the field of online social networks, collaborative recommendation systems (Yadav et al. 2021) are present on multiple platforms. For example, Facebook or Instagram systems recommend whom to follow, based on users’ friends or likes.
- Influence studies: These are based on obtaining the influence of brands, topics or people in certain sectors (Mora-Cantallops et al. 2021).
- Dissemination of information: In today’s information-saturated world, knowing the best way to disseminate information to reach more people is a critical factor. Recently, this sector has come under scrutiny due to the rapid dissemination of fake news or misinformation on topics of social interest such as COVID-19 (Himelein-Wachowiak et al. 2021).
- Privacy, security (Dadkhah et al. 2021) and truthfulness: This area focuses on the automatic verification of false accounts, identification of sources of spam as well as identifying the truthfulness of information (Cardinale et al. 2021) or identifying privacy violation issues.
- Opinion mining: A process by which we try to obtain relevant information from texts published on the web (Yousaf et al. 2020). This information can be, for example, polarity, orientation or relationships between text entities.

If we skip those problems that focus on graph theory and distance analysis, almost all of the problems addressed by social media mining, are related to text mining and Natural Language Processing. Text mining and Natural Language Processing aim to extract non-implicit knowledge from unstructured textual entities. Therefore, we could argue that social media mining is a specification of text mining, when text mining is applied to texts from websites or social networks. At this point, the technique diverges because it has to deal with problems inherent to user-generated content, such as colloquial expressions, emoticons, jokes or sarcasm.

Many of the solutions provided in the literature about social network mining involve supervised learning (Krawczyk et al. 2017; Noferesti and Shamsfard 2015; Cambria et al. 2010. But, what happens when the problem does not have any databases on which to train? This is the point where unsupervised methods, such as association rules, become relevant. Association rules are able to obtain in an unsupervised way co-occurrence relationships in large databases, such as those from online social networks. This means that association rules must be taken into account in social media mining problems because they provide fast, efficient and highly interpretable solutions, which are very valuable features in the decision-making process. Association rules, were created for transactional databases, therefore, to apply them in text problems, such as user-generated content, the first step would be to create the text transactions. We will study this in detail in the next section.

### Association rules for mining text from social networks

Association rules belong to the Data Mining field and have been used and studied for a long time. One of the first references to them dates back to 1993 Agrawal et al. (1993). They are used to obtain relevant information from large transactional databases. A transactional database could be, for example, a shopping basket database, where the items would be the products, or a text database, as in our case, where the items are the words, or more specifically, the entities represented by the words. In a more formal way, let ${t}=\{A,B,C\}$ be a transaction of three items *(A, B and C)*, and any combination of these items forms an itemset. In this case, all the possible itemsets are: $\{A,B,C\}, \{A,B\}, \{B,C\}, \{A,C\}, \{A\},\{A\}, \{B\} and \{C\}$. According to this, an association rule would be represented in the form *X*$\rightarrow$*Y*, where *X* is an itemset that represents the antecedent, and *Y* an itemset called consequent where ($X \cap Y = \phi$). As a result, we can conclude that consequent itemsets have a co-occurrence relation with antecedent itemsets. Therefore, association rules can be used as a method for extracting hidden relationships among items or elements within transactional databases, data warehouses or other types of data storage. The classical way of measuring the goodness of association rules regarding a given problem is using three measures: support, confidence and lift, which are defined as follows:

- Support of an itemset. It is represented as *supp (X)*, and is the proportion of transactions containing itemset *X* out of the total number of transactions of the dataset (D). Support is defined by equation (1).
  1 $$\begin{aligned} supp(X) = \frac{|{t\in D : X\subseteq t}|}{|D|} \end{aligned}$$
- Support of an association rule. It is represented as *supp(X *$\rightarrow$ *Y)* and is the total amount of transactions containing both itemsets *X* and *Y*, as defined in the following equation:
  2 $$\begin{aligned} supp(X \rightarrow Y) = {supp(X \cup Y)} \end{aligned}$$
- Confidence of an association rule. It is represented as *conf (X*$\rightarrow$*Y)* and represents the proportion of transactions containing itemset *X* which also contains *Y*. The equation is:
  3 $$\begin{aligned} conf(X \rightarrow Y) = \frac{supp(X \cup Y)}{supp(X)} \end{aligned}$$
- Lift. It is a useful measure to assess independence between itemsets of an association rule. The measure *lift (X*$\rightarrow$*Y)* represents the degree to which X is frequent when Y is present or vice versa. Lift is a very interesting measure as it relates the antecedent and the consequent through the concept of independence. A value of 1 indicates that the appearance of the consequent and the antecedent in the same rule is independent, therefore, the rule has no effect. On the other hand, lift values greater than 1 indicate a dependence between antecedent and consequent that will make the rule perfect for predicting the consequent in future datasets. Negative values indicate that the presence of one item has a negative effect on the presence of another. Lift is defined mathematically in the following way:
  4 $$\begin{aligned} lift(X \rightarrow Y) = \frac{conf(X \rightarrow Y)}{supp(Y)} \end{aligned}$$

Since association rules demonstrated their great potential to obtain hidden co-occurrence relationships within transactional databases, they have been increasingly applied in different fields. Among other fields, one in which association rules have attracted a lot of interest is Text Mining. One of the first papers which addresses the problem of text mining with association rules, is the paper presented by Martin-Bautista et al. (2004). In this work, textual transactions are defined, on which fuzzy association rules are applied. Textual transactions are necessary in order to be able to apply association rules on text, opening the possibility of applying association rules to text mining problems. In this field, text entities (opinions, tweets,...) are handled as transactions in which each of the words is an item. In this way, we can obtain relationships and metrics about co-occurrences in large text databases. Technically, we could define a text transaction as:

#### **Definition 1**

Text transaction: Let *W* be a set of items (words in our context). A text transaction is defined as a subset of words, i.e. a word will be present or not in a transaction.

For example, in a Twitter database, in which each tweet is a transaction, it will be composed of each of the terms that appear in that tweet. So the items will be the words. The structure will be stored in a term matrix in which the terms that appear will be labelled with 1 and those that are not present as 0. For example for the transactional database $D=\{t1,t2\}$ being $t1=(just, like, emails, requested, congress)$ and $t2=(just, anyone, knows, use,$ *delete*, *keys*) the representation of text transactions is shown in Table 1.

**Table 1:** Example of a term matrix in a database with two textual transactions

Transaction\Item	Anyone	Congress	Delete	Emails	Just	Keys	Knows	Like	Requested	Use
t1	0	1	0	1	1	0	0	1	1	0
t2	1	0	1	0	1	1	1	0	0	1

We can see how in a real problem this matrix will be very sparse. This is a problem that is often mitigated with different data cleaning processes. For example, in some cases, terms that are similar or represent a closely related entity are exchanged for this entity so that the term matrix is less sparse.

Transactions are an essential part of the association rule mining extraction process and without them we would not be able to mine frequent patterns. Association rules cannot be applied on raw text, so with this internal representation of text as textual transactions, association rules can be applied to almost any textual data problem. The internal representations of the matrix can also be calculated instead of using the absolute frequency, by employing its TF-IDF^2^, TF^3^ (Robertson 2004) or even its fuzzy membership value, which gives to this representation a great versatility still to be exploited. Having any of these internal representations for transactions, it is possible to apply any association rules mining approach.

The most widespread approach for mining association rules is based on the downward-closure property of support and consists of two stages. To be considered frequent, the itemset has to exceed the minimum support threshold. In the second stage, association rules are obtained using the confidence or other assessment measure. To obtain the frequent itemsets, the algorithms, based on a minimum support value, will generate all the possible combinations of itemsets and will check if they are frequent or not. In each iteration, all the possible different itemsets that can be formed by combining those of the previous iteration are generated, so the itemsets will grow in size. Within this category we find most of the algorithms for obtaining association rules, such as Apriori, proposed by Agrawal and Srikant Agrawal and Srikant (1994). Apriori is based on the premise that if an itemset is frequent, then all its subsets are also frequent. It means when we find one of unfrequent itemsets, all other itemsets containing this one do not need to be analyzed. Thus, we can prune the search tree avoiding checks and increasing efficiency. Although the most widely used algorithm is the Apriori algorithm, it is not the only one. We can find others such as FP-Growth proposed by Han et al. Han et al. (2000) and Eclat (Ogihara et al. 1997).

Eclat Ogihara et al. (1997) is an improved version of the Apriori algorithm, which improves the execution times of the algorithm. The main difference with the Apriori algorithm is that the Eclat algorithm performs a vertical search, similar to the depth-first search of a graph, as opposed to the breadth-first search performed by the Apriori algorithm. The basic idea is to compute intersections between items. To do this, a list of items is created, in which the items are related to the transactions in which they appear. With this list, the algorithm can compute the support value of a candidate itemset and avoid generating subsets that will not reach the support threshold. In this way, it can reduce the computation time in obtaining rules, but the management of this list implies a higher memory consumption.

The FP-Growth algorithm (Han et al. 2000) was proposed in 2000, as a solution to the memory problems generated by typical methods such as Apriori, seen above. It is a very efficient algorithm and is widely used in problems and solutions that could be framed under the name of Big Data. FP-Growth creates a compressed model of the original database using a data structure called FP-tree, which is made up of two essential elements:

- Transaction network: Thanks to this network, the entire database can be abbreviated. At each node, an itemset and its support are described and calculated by following the path from the root to the node in question.
- Header table: This is a table of lists of items. That is, for each item, a list is created that links nodes of the network where it appears. Once the tree is constructed, using a recursive approach based on divide and conquer, frequent itemsets are extracted. To do this, first the support of each of the items that appear in the header table is obtained. Second, for each of the items that exceed the minimum support, the following steps are carried out:
  1. The section of the tree where the item appears is extracted by readjusting the support values of the items that appear in that section.
  2. Considering the extracted section, a new FP-tree is created.
  3. The itemsets that exceed the minimum support of this last FP-tree are extracted.

Thus, FP-Growth requires less memory than Apriori. Additionally, the divide and conquer principle makes FP-Growth attractive in Big Data environments. It is worth noting that one of the reasons why FP-Growth is more efficient is that it is not exhaustive, i.e. it does not get all possible rules. Apriori, on the other hand, does (Fernandez-Basso et al. 2016).

Before finishing this section it is necessary to mention that the internal binary or fuzzy matrix representation is not the only possible way to represent the textual transactions. In some studies a standard representation is used in which the terms and texts present in social networks are used to form a new transactional database. For example, let us imagine a database of tweets. By means of the text mining procedure we can obtain feelings about each of the tweets and build a transactional element called sentiment, which could take the values sentiment=positive, sentiment=negative, sentiment=neutral. Another transactional element could be names, which would take the output produced by a Named Entity Recognition over the tweets. The Named Entity Recognition (NER) process is an automatic process that identifies entities and assings them a category within their grammatical or lexical category (Ritter et al. 2011). One of the most famous systems, proposed by Stanford University (Manning et al. 2014), can be used to obtain the names of people, places, etc. mentioned in a given document. With these textual categories located and tagged, we can create transactions. For example, names = (Katie) or names = (Biden,Trump). In this case a concrete example would be $D=\{t1,t2\}$ being $t1=(sentiment=positive, names=Trump)$ and $t2=(sentiment=neutral, names=Biden)$. On these two transactions we could perfectly apply association rules to find which feelings have more co-occurrence with certain names.

## Tasks

In this section we have grouped the studies that apply association rules according to their data mining task. In Sect. 5, we will analyse the fields or domains of application of these tasks. A summary of all of them has been added in Table 2.

### Summarization

The large amount of data present on the web has influenced the appearance of systems capable of presenting this information in a summarized form. Association rules have the potential to bring together words and terms by means of co-occurrences and frequent itemsets, therefore, this is one of the fields of social media mining in which association rules proliferate the most. r One of the first papers to apply association rules to summarization is Kacprzyk and Zadrozny (2003). Within this category, there are articles focused on summarizing information from Twitter. These articles are based on summarizing threads of conversation on a particular topic, so that the reader can get a reliable and complete idea quickly. In this area we also found the proposal in Phan et al. (2018) that uses maximal association rules to summarize Obama’s most important tweets.

### Topic detection

Topic detection is based on the ability of an intelligent system to detect what is being talked about, or what a specific discussion in a social network is about. That is, they try to tag a set of textual entities according to a topic. In this sense, probabilistic and unsupervised techniques such as LDA^4^ (AlSumait et al. 2008; Jelodar et al. 2019), stand out, but given the unsupervised nature of the problem we also find some approximations by association rules.

One of the first studies in this line dates from the year 2000 with the work described in Ramamonjisoa et al. (2000). This paper focuses on obtaining new research topics on the text in the WWW^5^. To do so, it uses association rules obtained from the keywords of the publications. A more recent study in this line is the paper by Cagliero and Fiori (2012), where a solution through generalised association rules is offered, which provides a compendium of topics used in the social network Twitter. This paper uses dynamic association rules that are generalized through the context of the posts and the content of the tweets generated by the user. In Mai et al. (2020) Mai et al. use association rules to build a Twitter data analytics application that allows to a hypothetical user to find out what is being discussed about their profession on Twitter. The system is based on the Apriori algorithm and data visualisation, so a non-technical user could use it. To test how well it works, the system was tested on a real case with physician assistants.

In the field of topic detection, association rules stand out when being used to obtain knowledge about a specific topic, for example, about cyber bullying (Margono et al. 2014; Zainol et al. 2018). In this way, the authors use the Apriori algorithm to obtain concrete patterns which helped to identify topics in social networks used for cyber bullying. That is, on a specific topic, association rules are used to obtain subtopics or more detailed information and knowledge. In the same direction as the previous ones, but oriented to tweets from insurance, we find the study proposed by Mosley Jr (2012).

As for topic detection, but in reviews on digital banking of a Google Play Store app, we found the paper (Cheng et al. 2020). In it, Cheng and Sharmayne propose to use LDA and association rules in conjunction. They first obtain topics about the reviews, and create two clusters depending on whether they are positive or negative. Once this is done, they obtain association rules that relate terms from each topic to one of the clusters. With the result, they try to obtain which words in the customer reviews are related to positive or negative concepts of the services of the bank’s app.

### Event detection

Event detection is closely linked to the detection of topics, but characterized by having a temporal character, for example, the identification of possible terrorist attacks, floods or earthquakes. In this area of application association rules are playing a major role again due to the possibility of using them without prior training. In this sense, they are very appropriate and robust to be used in data from unfiltered social networks and processing them in real time.

Two papers that are related according to the data set used are the one proposed by Adedoyin-Olowe et al. (2016) and Fernandez-Basso et al. (2019). Both try to detect events in politics and sports events. The first addresses the problem by matching rules on hashtags, whilst the second is a spark-based distributed solution that improves detection trends in the form of frequent itemsets without obtaining rules.

For events related to possible problems or catastrophes, we find the papers (Fu et al. 2015; Huizinga et al. 2017; Acosta and Palaoag 2019; Rodavia et al. 2018). All of them make use of a crawler on websites and social networks to obtain event detection patterns on transport and traffic in the first case, insurance and natural disaster in the second and third and on flood areas in the Manila underground in the last case. These studies try to exploit the potential of association rules to obtain relevant patterns quickly on collected data by the crawler in real time. However, these systems are unreliable compared to other systems based, for example, on classification. Supervised systems are most suitable for event detection problems, especially when human lives may be at stake. In the field of health, and particularly in monitoring possible health-related behaviour events on social networks, we find the paper (Jung et al. 2016). In it, association rules are used to monitor health in Korea to face the problem of yellow dust.

A paper that is very interesting due to the nature of the used data is the one proposed by Zhang et al. (2013). This paper tries to detect events on video and uses adaptive association rules on video metadata and video tags. The obtained patterns are used to feed an event classification system that also uses a Near-Duplicate Keyframe identifier.

### Sentiment analysis

For the application of association rules for sentiment analysis, there are also some recent hybrid approaches that use association rules in conjunction with other techniques. Hybrid approaches can be defined as proposals that use association rules and other techniques (usually classification algorithms) to improve the results. In these cases, due to the nature of the problem, association rules cannot be used alone, but they can be used to improve the results of later stages of classification. Association rules play a role in finding relationships between terms that are grouped by patterns in the classification stage, improving the results to a significant degree.

In this case we find two papers (Zainuddin et al. 2018; Dehkharghani et al. 2014) where a hybrid approach is proposed using association rules to generate co-occurrences of terms related to feelings and thus create a much more powerful system of sentiment analysis. The first paper uses a dataset of crimes and motives for crime, which is compared with methods such as PCA^6^, showing that the method using the rules for co-occurrences improves what already exists. The latter paper (Dehkharghani et al. 2014) applies association rules in an earlier stage of sentiment classification, summarizing the debates about Kurds on Twitter.

Closely linked to the above hybrid models but in the health field we find the paper (Paulose et al. 2018). In this, the authors use association rules mining and natural language processing techniques to mine the social network Twitter for the use of Fentanyl. After a process of sentiment analysis they use association rules to obtain the correlations of its use with other drugs and products of a dangerous nature for health.

To conclude this section, it is necessary to mention a paper that offers a version of sentiment analysis based only on association rules. This is the paper (Diaz-Garcia et al. 2019), in which the authors generalize the association rules obtained by the majority sentiment, cataloguing the emotions around a politician on Twitter.

### Forecasting

Due to their social nature, online social networks have been the subject of forecasting studies since their creation. This is because in many social networks and websites the data is public, and people have opinions about certain issues. These opinions and posts can be used for prediction systems in various aspects such as politics or studies of influence on a certain sector. In this latter case these studies try to report which users are more influential in a certain network with the aim, for example, of being used as seeds for spreading marketing or publicity strategies.

In this area of application we find the papers by Erlandsson et al. (2016b), Erlandsson et al. (2016a). In Erlandsson et al. (2016b) they propose a system based on association rules to identify which users are more influential, pursuing the idea of comparing the system with the state of the art in the field, with these being the Page Rank Centrality and the Degree Centrality. The results are promising, although they highlight a problem of execution time.

On the other hand, in the paper (Erlandsson et al. 2016a) they predict, using association rules, the participation of users in Facebook, something useful that could be used for example to contact certain high participation users for possible promotions. In line with Facebook, the paper by Nancy et al. (2013) uses the Apriori algorithm on a data set of universities to study the influence of gender studying a course.

In the Twitter domain the papers (Abu Daher et al. 2018; Daher et al. 22018) use hashtags that are trending topics, that is, those hashtags used by a several number of tweets in a concrete moment, as textual entities that feed the association rule mining algorithm. With this, they obtain patterns from the most popular users of the social network at that certain moment.

Also in Twitter, the paper (Diaz-Garcia et al. 2020a) is oriented to discern patterns of fake news in a Twitter data set about the 2016 American presidential elections. The paper (Diaz-Garcia et al. 2020b) proposes a solution based on big data capable of cataloguing and obtaining knowledge in an unsupervised way. The system is tested with a set of more than 1M tweets obtained from the United States, specifically, tweets of a political nature. In all the studies, solutions are provided that conclude in the potential of the use of association rules to undermine social networks for voting predictions. n the field of fake news and hoaxes forecasting, we also find the paper (Utami et al. 2020) where Utami et al. create a classifier based on Random Forest to discover hoaxes on Twitter. At this point, the association rules take part through the Apriori algorithm, used to discover associations between words that simplify the learning process. Again, this is a hybrid approach, where the frequent pattern discovery potential of association rules greatly enhances the results of classification-oriented algorithms.

Tourism is one of the areas where forecasting is more relevant. In this sector, we find the paper (Chugh and Phumchusri 2020) that uses the TripAdvisor social network to train rating models from 1 to 5 stars. Within each of the models the association rules are used to see which terms are more related to others, to know which words are the ones that cause an opinion to be better or worse.

In the field of smart cities, we find the paper (Shen et al. 2018), where the authors use Twitter and spatio-temporal pattern mining with an adaptation of the apriori algorithm to determine which roads in a city are likely to be most congested at a certain time.

We conclude this section by discussing crime surveillance. Crime and criminal organisations are very present in social networks. Having systems capable of dealing with this scourge and giving early warnings is very much needed by law enforcement agencies. In the fight against these problems we find the paper (Tundis et al. 2019). This paper uses a compendium of data mining techniques to trace the similarity between users that may be related to crimes. To this end, the paper uses association rules and other techniques such as clustering.

### Collaborative social systems

Data from social networks as well as user actions can be used to generate collaborative systems that improve the experience of other users. These systems exploit the premise that if something has been useful for a user, it will also be useful for a user with a similar profile. Under this premise, we find different approaches such as collaborative recommendation systems, collaborative expert systems or social tagging systems. Collaborative recommendation systems aim to be able to recommend items (films, songs...) to certain profiles based on their similarity to other profiles. They are based on the recommendations or ratings given by certain users, so that these preferences enrich the recommendation system. As for expert systems, the system simulates human reasoning in order to make decisions. In this case, certain actions or considerations offered by the system may be motivated by similarity to the situation, or requirements. Finally, social tagging systems try to recommend tags for certain items, based on the tags that another profile has left on similar items. In all three cases, there is an underlying functionality, which is the need to obtain common patterns between different items or profiles. That is why these elements are studied together at this point.

#### Recommendation and expert systems

In the field of collaborative recommendation and expert systems we find quite a few studies that make use of association rules. This is because the nature of association rules for finding co-occurrence relationships is one of the ways in which expert and recommendation systems are developed internally.

Two closely related papers that use association rules to obtain Twitter patterns that are related and can be used in a later expert system are (Mamgain et al. 2016) and Das et al. (2019). The first deals with the problem of choosing a school, for which it obtains patterns of good schools and terms related to them from the social network Twitter. Along the same lines, but to promote cycling, the paper (Das et al. 2019) extracts patterns that relate good habits to promote the population to use bicycles. Also in the field of health, we find the study (Hamed et al. 2014) that seeks to get patterns on Twitter that could be used by other people to give up smoking.

As far as recommendation systems are concerned, we found some important papers that make use of the rules for recommendation systems ranging from schools to movies as well as mobile services or courses (Kakulapati and Reddy 2019; Xin et al. 2020; Huang et al. 2018). In the paper (Kakulapati and Reddy 2019) the authors use the Apriori algorithm to relate actors and metadata from films to other similar films. It is based on the premise that related films will be a good recommendation for the users. The underlying background in the other papers is very similar but applied to suggest mobile services and courses respectively. The paper (Si et al. 2019) uses Twitter and Linkedin to create a system capable of relating users preferences to the most acceptable job applications for them. All this is possible by using just text mining techniques and association rules.

In Zheng (2020) authors apply an extended version of the Apriori algorithm to a health shopping website. The purpose is to recommend products to users based on the relationship of some products with others, as well as the user’s behaviour with respect to certain products. Although the results are promising, the authors conclude that the large amount of data present in these databases limits the use of Apriori, so new and more efficient versions should be explored.

Finally, in Rao et al. (2019) Rao et al. propose an unsupervised recommendation system based on the Apriori algorithm and Named Entity Recognition. The system is designed to analyse trends on Twitter, and recommend tweets related to the context of each one in order to consolidate an opinion on them. The system first obtains named entities and then extracts frequent patterns among them. These patterns are represented in trees, having in the leaf nodes the tweets related to the context of a given trend.

#### Social tagging systems

Finally, it is necessary to talk about social tagging at this point. Social tagging is based on the possibility of tagging through the community of users, any online resource, being it text, movies or music. These tags become part of the communities domain, facilitating thus the search for a certain resource. At this point, association rules have a quite obvious role, since they offer the user words that are largely related to the ones they use as labels, favouring and enriching the system (Kammergruber et al. 2010; He et al. 2010). Another study in this line proposed by Feng, et al. (2010) is very interesting because it exploits the relationship of emotions and colour with social tagging through association rules. In this study, association rules are used over an encoded image, associating pixels of a colour to certain emotions, something that shows that the use of association rules in social media mining, has innumerable and creative applications.

## Fields of application

**Table 2:** Papers using association rules according to their task and field

Paper	Task	Social Network	Academics	Crime	Health	Insurance	Influence	Leisure	Natural disasters	Politics	Sports	Transport	Trends
Phan et al. (2018)	Summarization	Twitter								X			
Ramamonjisoa et al. (2000)	Topic Detection	Web posts	X										
Cagliero and Fiori (2012)	Topic Detection	Twitter											X
Margono et al. (2014)	Topic Detection	Twitter		X									
Zainol et al. (2018)	Topic Detection	Twitter		X									
Mosley Jr (2012)	Topic Detection	Twitter				X							
Mai et al. (2020)	Topic Detection	Twitter	X		X								
Cheng et al. (2020)	Topic Detection	Google reviews						X					
Adedoyin-Olowe et al. (2016)	Event Detection	Twitter						X		X	X		X
Fernandez-Basso et al. (2019)	Event Detection	Twitter						X		X	X		X
Fu et al. (2015)	Event Detection	Twitter										X	
Huizinga et al. (2017)	Event Detection	Twitter				X			X				
Acosta and Palaoag (2019)	Event Detection	Twitter							X				
Rodavia et al. (2018)	Event Detection	Twitter							X				
Jung et al. (2016)	Event Detection	Twitter			X								
Zhang et al. (2013)	Event Detection	Youtube		X					X	X	X		X
Zainuddin et al. (2018)	Sentiment Analysis	Twitter		X									
Dehkharghani et al. (2014)	Sentiment Analysis	Twitter								X			
Paulose et al. (2018)	Sentiment Analysis	Twitter			X								
Diaz-Garcia et al. (2019)	Sentiment Analysis	Twitter								X			
Erlandsson et al. (2016b)	Forecasting	Twitter					X						
Erlandsson et al. (2016a)	Forecasting	Facebook					X						
Nancy et al. (2013)	Forecasting	Facebook	X				X						
Abu Daher et al. (2018)	Forecasting	Twitter					X						X
Daher et al. (22018)	Forecasting	Twitter					X						X
Diaz-Garcia et al. (2020a)	Forecasting	Twitter								X			
Diaz-Garcia et al. (2020b)	Forecasting	Twitter								X			
Utami et al. (2020)	Forecasting	Twitter								X			X
Tundis et al. (2019)	Forecasting	Twitter		X									
Chugh and Phumchusri (2020)	Forecasting	TripAdvisor						X					
Shen et al. (2018)	Forecasting	Twitter										X	
Mamgain et al. (2016)	Expert System	Twitter	X										
Das et al. (2019)	Expert System	Twitter			X							X	
Hamed et al. (2014)	Expert System	Twitter			X								
Kakulapati and Reddy (2019)	Recommendation	Web Posts						X					
Xin et al. (2020)	Recommendation	Web Posts						X					
Huang et al. (2018)	Recommendation	Web Posts	X										
Si et al. (2019)	Recommendation	LinkedIn	X					X					
Rao et al. (2019)	Recommendation	Twitter											X
Zheng (2020)	Recommendation	Web Page			X			X					
Kammergruber et al. (2010)	Social Tagging	Web Tags						X					
He et al. (2010)	Social Tagging	Web Tags						X					
Feng, et al. (2010)	Social Tagging	Web Tags						X					
		**Total**	6	5	6	2	5	11	4	9	3	3	8

In this section, we will go into detail on the field of application where association rules have been applied in the social media mining domain. We must take into consideration that we have gathered the fields in large groups, but they are not exclusive or the only ones, simply those with enough scientific weight for being able to categorize them. The fields of application have been distilled from the reading of the papers and the dataset used:

- Academic: In this area, we have included those papers that deal with the academic or professional world. For example, those in which a university recommendation is proposed or association rules are used to obtain information on new research fields.
- Crime: In the area of crime we have included all those actions catalogued as against the law, from theft to cyber bullying, as well as more serious crimes such as homicides.
- Health: Everything related to health, from epidemics prediction to patterns detection, useful for recommendation systems such as for giving up smoking, for example.
- Insurance: Everything related to insurance, which is closely linked to other branches such as transport and natural disasters, so we will have overlapping papers in these areas.
- Influence: In this area we have included influence studies either of users or topics, linked with trends. This can be catalogued as a kind of sub-section in forecasting where the central issue is to find who or what is really influential on the web.
- Leisure: This area encompasses leisure time, news, e-commerce, digital banking, and tourism actions that can be taken to spend time on social networks and the internet.
- Natural Disasters: Floods and disasters generally influenced by the weather and about which people post their warnings and alarms on social networks.
- Politics: This area includes news about politicians, datasets on opinions and hashtags related to electoral processes or tweets and posts released by a politician.
- Sports: Related to football matches or any other type of sports competition.
- Transport: This relates to transport, either personal, citizen mobility or professional related to logistics.
- Trends: Trend analysis is one of the most studied areas in data mining applied to social networks. A trend would be that which at a particular time is heavily posted on social networks, it can be related to many topics, even a cluster of them, but must be linked to a moment or several moments in time.

For a better understanding, Table 2 comprises a compilation of the references cited above. Also Table 2 shows the application, the social network used and the field of application where they were applied. If we analyse the table, we can see how many papers are transversal, that is, they exploit more than one field of application. This shows that association rules are versatile and there are many fields in which they can be used. One of the reasons for this is linked to their potential for interpretability. This factor is very relevant today, as all areas are asking for explainable and interpretable Artificial Intelligence systems, that are easier to understand contrary to other black box systems. This is undoubtedly a great potential in descriptive problems or previous data analysis. Furthermore, association rules cannot be considered as an alternative to other supervised techniques. This is one of the reasons why the revised works are focused on descriptive analyses and when they are predictive, association rules are used in conjunction with other supervised techniques. Finally, we can see how the field of politics and leisure is currently arousing a lot of interest. In this point, association rules stand out considerably because of their potential to correlate terms or users with certain policies or parties, for example.

## Current challenges and future trends

As we have seen in the previous section, association rules are a very interesting tool to address the problem of social media mining when, due to restrictions or needs of the problem, it is necessary to deal with it in an unsupervised way. On the other hand, there are still some challenges that are already being addressed and that undoubtedly open up a line of future trends to be faced in the coming years in the field of association rules and social media. The most relevant of these challenges are:

- Streaming association rules: Although association rules were created to address the problem of data mining in large transactional databases, the paradigm has mutated and they are now required in streaming environments. Currently, one problem that has been solved is that of discovering frequent itemsets in streaming (Calders et al. 2007; Jin and Agrawal 2005; Xie and Tan 2019). Like for association rules, there are some incipient works about this (Abd Elaty et al. 2018; Xiao et al. 2011). Many of these articles name the process of obtaining association rules but they are really based on approximations or data structures for obtaining frequent itemsets. The main problem in obtaining association rules is that in traditional databases the algorithms can take several passes adjusting supports according to thresholds. This is unfeasible in streaming because old data has to be discarded and the supports have to be readjusted according to the time windows. The main concern in addressing this solution is to provide an efficient data structure capable of storing and calculating support and fitting measurements correctly over time and the arrival of new data. There is some work already in this line of research (Liu et al. 2021) but it still needs to be tested and used in problems related to the domain of social networks user-generated text, where to our knowledge, there are still no systems applied to solve this particular problem. Therefore, we consider it to be a current line of research and a current challenge.
- Temporal association rules: This is a similar idea to streaming but can be applied over association rules in a standard transactional database. In it, we would have time stamps in each transaction. The obtained rules could be compared in time and would be very relevant in problems of influence detection, because something that today is influential can stop being so in a posterior period of time. There are already some proposals that use temporal fuzzy association rules aiming to address this challenge (Chen et al. 2020).
- Socialized association rules: Generalized association rules are a great tool to improve the performance of association rules in environments with a large dispersion of items, such as social media environments. This technique was introduced by Srikant and Agrawal Srikant and Agrawal (1997) in 1995. They propose that the rule *{Strawberries, Oranges} *$\rightarrow \{Milk\}$ could be replaced by $\{Fruit\} \rightarrow \{Milk\}$. This hierarchical point of view allows a higher level of abstraction that offers the possibility of obtaining even more information from our data. Creating a topology of generalized association rules by social flags could be a great solution to problems such as event or topic detection. In this area there are already some studies. On the one hand, the research Diaz-Garcia et al. (2019) tries to extend and generalize association rules by feelings. On the other hand, the proposal in Wang et al. (2020) extends the rules using graph theory, something that could have innumerable applications in online marketing. This point also offers the possibility of enrichment by other elements. For example, for a crime detection application in social networks it would be very interesting to enrich and extend the rules or frequent terms by means of police databases.
- Data dispersion: One of the main problems with user-generated text association rules in online social networks is the granularity and dispersion of terms. That is, when the domain under study occurs in Twitter or TripAdvisor, it is very complicated for a term to appear several times in a post. Therefore, the internal binary representation for transactions should be very smooth for dispersed data. Finding an internal representation with weights and capable of capturing the nature of the analysis in the matrix will offer great results. In this context, there is a large research line in the problem of sparse matrices (Jiang et al. 2020; Demirci and Aykanat 2020; Zhang et al. 2020). But an appropriate solution for matrices obtained from social networks is still missing.

## Discussion

In this paper, we demonstrated the great potential of unsupervised techniques and, more specifically, of association rules to deal with user-generated text mining problems in social networks. Regarding the strengths and weaknesses of association rules as they apply to user-generated content, it should be noted that they are inherent to the technique and are common across the different fields of application. The greatest strength of association rules in the field of social network text comes from their capacity to provide information. The output of the algorithms is easily interpretable and extrapolated across all the application domains seen. This facilitates their use by non-technical users. We have also detected that association rules are used because of their potential to summarise information and relate certain textual components with others that would be difficult to see explicitly. These relationships allow a better understanding of the problem domain, for example which disease is related to which medicine or which city is related to which tourist attraction. In this way, terms with little relationship can be avoided, favouring the analysis of what is really necessary. Finally, it should be noted that the unsupervised potential of association rules makes them perfect candidates for a first stage of data mining on which to build hybrid models with both supervised and unsupervised components. As for their weaknesses in the field of their application to social network texts, it should be noted that the textual transaction matrix is very sparse, which makes it difficult to load in memory. Also, it is a technique that is very sensitive to lies and colloquial expressions in social networks. It is clear that its potential is greater than its weaknesses and that is why interest in this subject has grown considerably in the last years.

**Fig. 1:**
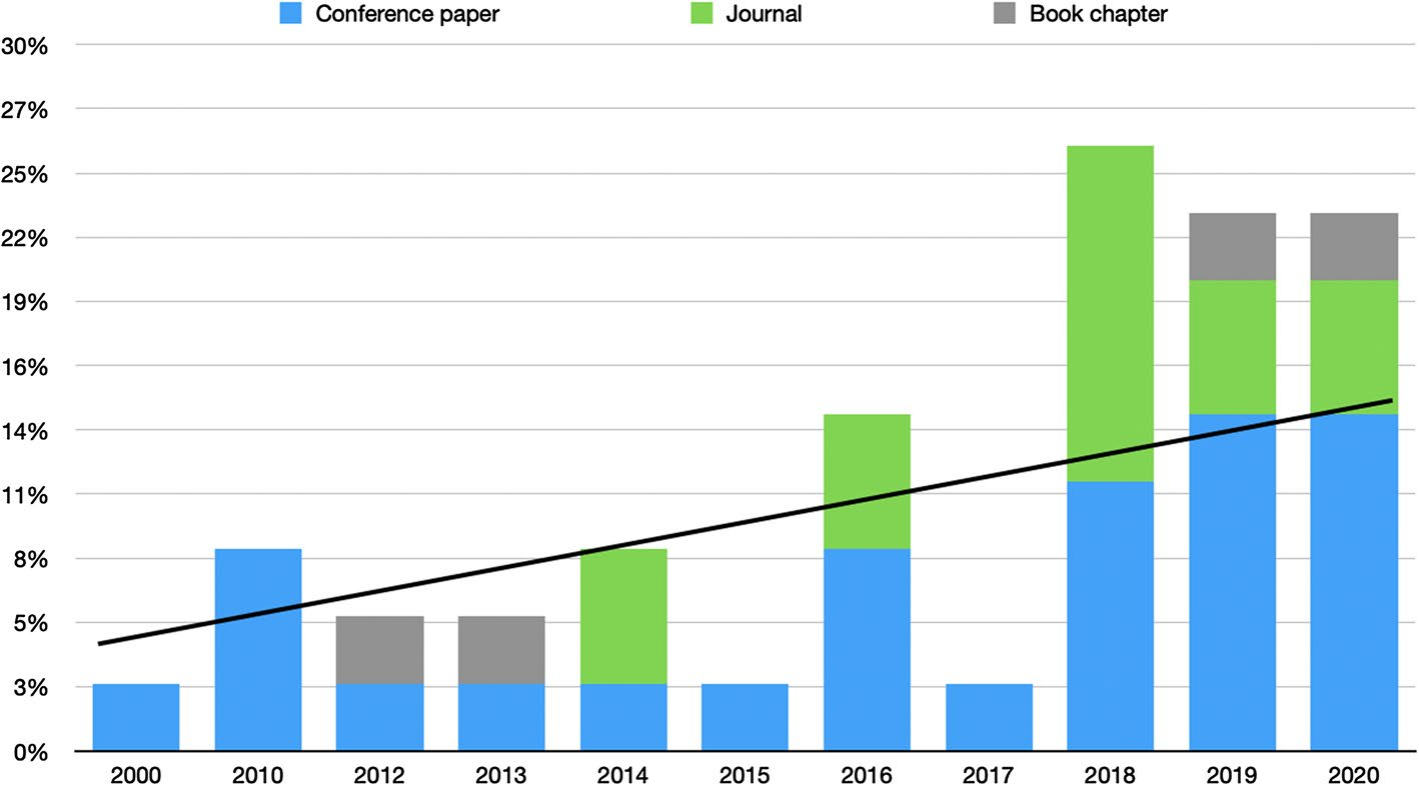
Papers according to their type and year of publication in social media mining

If we look at the publication years (Fig. 1) we can see how the publication trend is increasing. This is due to the fact that social networks have a very recent period of assimilation that starts in 2010 and onwards, so in the first years the number of papers is lower. On the other hand, in recent years the number of papers has increased reaching almost 75% of the total number of papers in the last 3 years. This is due to two reasons. Firstly, it is now when the social networks have a very relevant role in our lives. Secondly, association rules are arousing interest in applications related to social networks given their descriptive potential and interpretability. This is undoubtedly a warning that in the coming years the number of publications such as those contained in this survey will increase considerably, especially those in the health field marked by the COVID-19 pandemic. The pandemic is arousing a lot of interest in the field of artificial intelligence and more specifically in the field of social networks. In this field, we can find numerous papers that deal with sentiment analysis on prevention or social distancing measures (Chintalapudi et al. 2021; Shofiya and Abidi 2021). Regarding association rules and COVID-19, we can see how there is a current application and research trend that uses the great interpretability of association rules to obtain patterns and relationships between symptoms (Tandan et al. 2021) or variants of COVID-19 and places (Atsa’am and Wario 2021). Based on user-generated content, it is only a matter of time before new articles can be added to the review. This can be seen from the fact that there are already some pre-prints, for example (Drias and Drias 2020), that attempt to address the scope of Twitter conversations related to COVID-19. On the other hand, the incursion of COVID-19 also increases other problems present in social networks, such as the dissemination of misinformation or fake news. This problem is also being addressed by techniques based on pattern mining. In Kasseropoulos and Tjortjis (2021) association rules are used in conjunction with a named entity detection process to discern if there is any pattern in the use of named entities in real news versus fake news. This article shows the growing interest and usefulness of association rules in conjunction with other less interpretable techniques. This is undoubtedly a symptom of the good health of association rules in the digital world.

Another important factor of the review can be outlined if we look at the kind of publication. The number of publications is around 90% in journals and conference papers. The remaining 10% are book chapters. The 90% is divided in a 56% of conference papers and a 44% of journal articles. Here we can see how journal publications, which normally have more impact, are almost entirely situated in recent years, which again leads us to think about the health and strength that association rules hold in the current academic community.

**Fig. 2:**
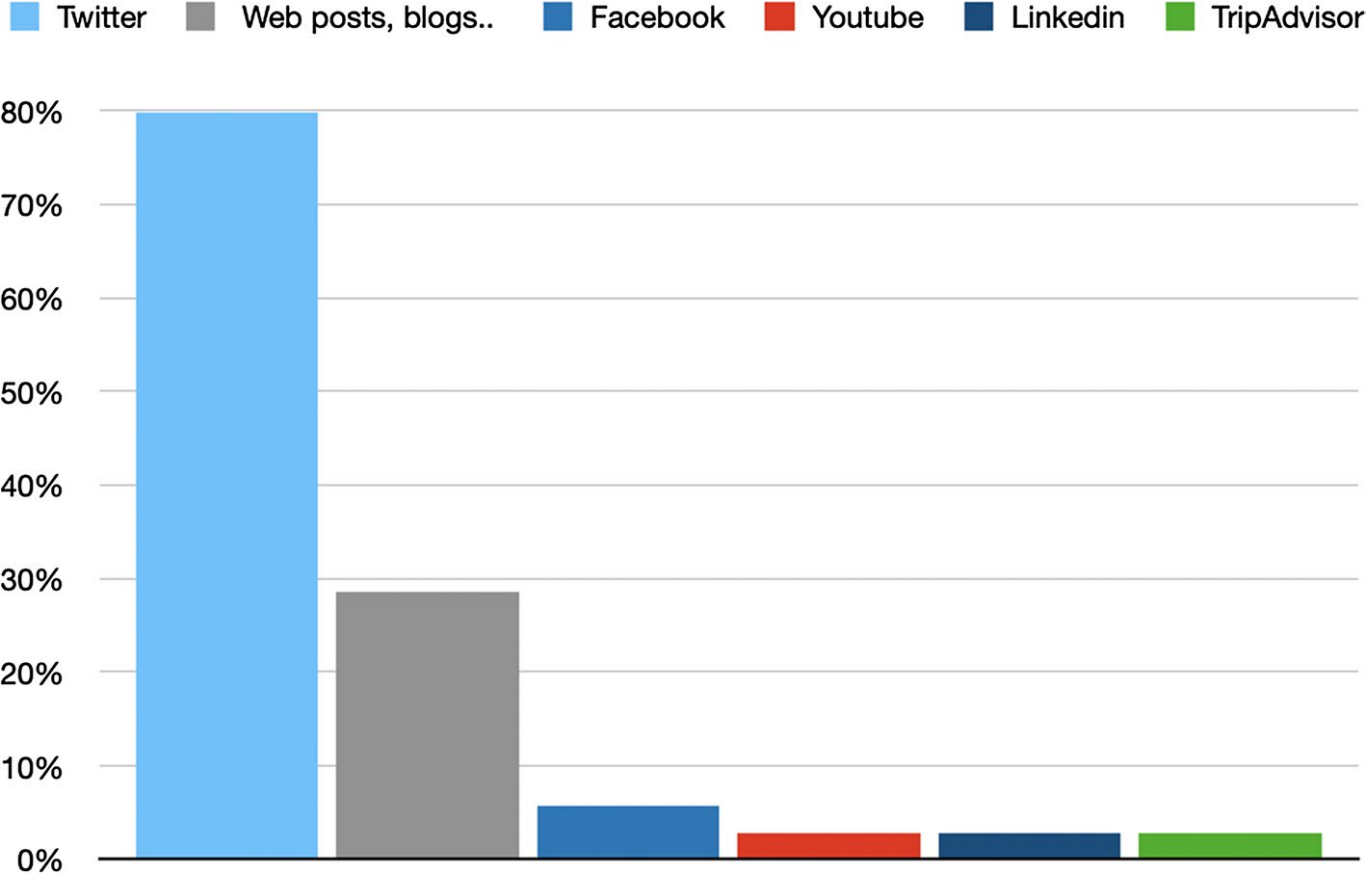
Papers according to their type of social networks data

Regarding the type of data, as we can see in the Fig. 2, Twitter continues to be the most widely used social network for data mining applications and work. This is because it offers APIS to obtain data freely and therefore it is very easy to obtain information about accounts or user-generated content that can feed these systems. Although Twitter stands out the most, it is interesting to mention how association rules are being used in very interesting applications that take into account networks as disparate as Facebook, YouTube, Linkedin or TripAdvisor.

## Conclusions

User-generated text in social media provides a great opportunity of knowledge as long as we are able to extract it correctly. We have demonstrated how association rules are necessary in social media mining problems. Following the analysis carried out, we can conclude that, at this moment, the power of the association rules lies in the interpretability of the model and the results.

Recently, explainable Artificial Intelligence is becoming increasingly relevant to business processes, as other black box solutions, such as neural networks, offer solutions that are difficult to interpret and maintain. Association rules offer a great interpretability of the results and the model, which makes them very easily extendable to business processes that have to deal with content from social networks. This is very important because these results will probably not have to be interpreted by a data engineer but could be interpreted by someone with less technical knowledge. In addition to interpretability, the potential of association rules in social media mining solutions framed in Big Data lies in the possibility of making use of pattern mining algorithms without having pre-labelled databases, i.e. without prior training. This offers the possibility of having systems capable of responding with sufficient speed to the volatility of social networks. Other models, such as those based on deep learning, need pre-trained networks for being able to respond quickly. Having these pre-trained networks is not trivial, and a change of context or trends in the networks would invalidate it completely. This offers a competitive advantage to association rule-based systems as they can adapt to these changes without the need for training. However, association rules cannot be positioned as an alternative to neural networks or other classification techniques in certain problems such as predictive or classificatory problems, as association rules in these cases may be not suitable.

In this paper, we have seen a lot of problems that are currently addressed using association rules, as well as their future challenges. With this in mind, we have tried to bring together the current knowledge of these techniques and their applications in different fields of textual entities present in social media. n the current research work, we have read, studied and classified several papers related to association rules in the field of social media mining. Our results indicate that the use of this technique is robust. Finally, we could summarize the conclusions obtained in this paper, through the answers to the research questions that we introduced in Sect. 1.

- RQ1: According to Sect. 4 the most relevant tasks implemented by association rules are summarization, event and topic detection, sentiment analysis, forecasting and collaborative social systems. It is necessary to point out that these may not be the only applications, but they are the most relevant ones, or at least the ones that bring together a considerable number of articles.
- RQ2: As we have seen in Sect. 5, there are several fields of application where association rules have been applied. These areas are: academic, crime, health, insurance, influence, leisure, natural disasters, politics, sports, transport and trends.
- RQ3: The most relevant future problems to be achieved by association rules are the streaming association rules, the temporal association rules, and the extended association rules by social flags or graphs (Wang et al. 2020), as pointed out in Sect. 7.

## Footnotes

1 Journal Citation Reports (impact factor).

2 Term Frequency - Inverse Document Frequency

3 Term Frequency

4 Latent Dirichlet Alocation

5 World Wide Web

6 Principal Component Analysis
